# Accuracy of Height Estimation Among Bystanders

**DOI:** 10.5811/westjem.2018.5.34877

**Published:** 2018-07-26

**Authors:** Sara Carey, Michaeleena Carr, Komaira Ferdous, Gina Marie Moffa, Jennifer Axelband, Shaila Quazi

**Affiliations:** * Aria Health, Department of Family Medicine and Emergency Medicine, Philadelphia, Pennsylvania; † St. Luke’s University Hospital and Health Network, Department of Critical Care Medicine and Emergency Medicine, Bethlehem, Pennsylvania; ‡ Aria Health, Department of Emergency Medicine, Philadelphia, Pennsylvania

## Abstract

**Introduction:**

High-risk mechanisms in trauma usually dictate certain treatment and evaluation in protocolized care. A 10–15 feet (ft) fall is traditionally cited as an example of a high-risk mechanism, triggering trauma team activations and costly work-ups. The height and other details of mechanism are usually reported by lay bystanders or prehospital personnel. This small observational study was designed to evaluate how accurate or inaccurate height estimation may be among typical bystanders.

**Methods:**

This was a blinded, prospective study conducted on the grounds of a community hospital. Four panels with lines corresponding to varying heights from 1–25 ft were hung within a building structure that did not have stories or other possibly confounding factors by which to judge height. The participants were asked to estimate the height of each line using a multiple-choice survey-style ballot. Participants were adult volunteers composed of various hospital and non-hospital affiliated persons, of varying ages and genders. In total, there were 96 respondents.

**Results:**

For heights equal to or greater than 15 ft, less than 50% of participants of each job description were able to correctly identify the height. When arranged into a scatter plot, as height increased, the likelihood to underestimate the correct height was evident, having a strong correlation coefficient (R=+0.926) with a statistically significant p value = <0.001.

**Conclusion:**

The use of vertical height as a predictor of injury severity is part of current practice in trauma triage. This data is often an estimation provided by prehospital personnel or bystanders. Our small study showed bystanders may not estimate heights accurately in the field. The greater the reported height, the less likely it is to be accurate. Additionally, there is a higher likelihood that falls from greater than 15 ft may be underestimated.

## INTRODUCTION

Trauma from falls is an important cause of both morbidity and mortality in children and adults. High-level falls (>15 feet [ft]) are a source of blunt trauma that can be difficult to evaluate and are characterized by multiple injuries across different body areas. Falls are the most common cause of admission to the emergency department during childhood and are the fourth leading cause of trauma deaths.^1^ Multiple trauma resulting from a high-level fall requires laborious investigation. To date, there are no current data to evaluate how closely heights are estimated by those at the scene of a fall.

Currently, emergency medical services’ (EMS) guidelines use fall height estimation as a criterion to determine the disposition of a patient to a trauma center or closest non-trauma center. The current height referenced as an indication for transfer to a trauma center is a fall from 20 ft for an adult and 10 ft for a child, or three times the height of the child.^4^ Most trauma centers and prehospital personnel use these guidelines to set trauma team activation protocols as well, determining the resources made available to the patient upon arrival and how quickly the patient is evaluated by the trauma team.

Demetriades et al. in 2005 evaluated injury patterns in falls from > 15 ft and found a higher rate of spinal injuries among patients over 14 years of age. This study also showed a range of mortality from 5.5% in the pediatric population to 24.3% in those over 65 years old.^1^ For adults, trauma from falls is associated with alcohol use in more than half of cases, and has a male predominance.^5^,^13^,^14^ The injuries sustained in adults from falls from a height vary from those of children, as adults tend to suffer axial loads from landing with their feet on the ground. Because of this, the most common injuries in adults tend to be fractures of the spine and lower limbs, particularly calcaneal fractures, and the most common spinal injuries tended to be in the lumbar region.^6^ Aside from skeletal injuries from falls, soft tissue injuries are also prevalent, the most common being brain injuries, followed by liver and lung injuries.^15^

**Figure 1:**
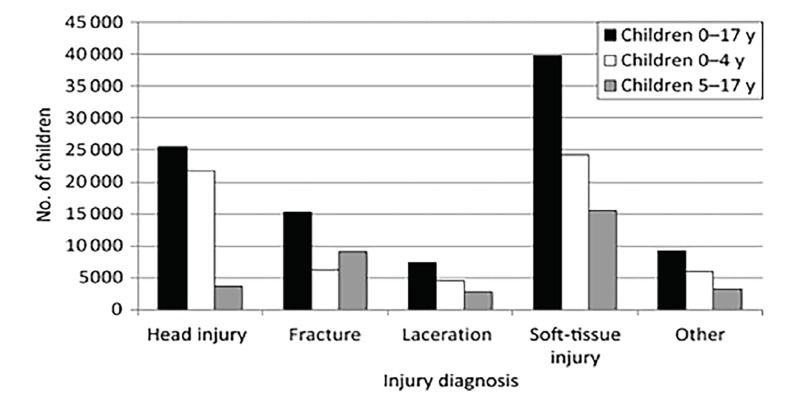
Distribution of pediatric injuries from falls.^18^

Trauma continues to be the most common cause of death in the U.S. pediatric population. In pediatric populations, high-level falls show a predominance of head and soft-tissue injuries as demonstrated in Figure 1.^18^ Another small study of 70 patients showed that 100% of children who fell from a height of two stories or fewer survived, but the mortality increased in falls from fifth- and sixth-story heights.^2^

Computed tomography (CT) imaging of head-injured children after a fall can carry a risk of radiation-induced malignancy. To identify children at very low risk of clinically-important traumatic brain injuries, for whom CT might involve unnecessary radiation exposure, the *Pediatric Emergency Care Applied Research Network* (PECARN) clinical decision tool is often used. Part of this tool incorporates the height of the fall. A severe mechanism is considered a fall of five ft for children over two years old and three ft for children under two years old.^9^ With regard to blunt abdominal trauma, PECARN clinical decision rules considered a height of 10 ft a severe mechanism.^21^

**Population Health Research Capsule**

What do we already know about this issue?

The height of a fall is considered relevant mechanistic information for trauma triage and evaluation; it is typically provided by prehospital personnel or bystanders.

What was the research question?

How accurate are height estimations in the absence of reference points (such as a storied building)?

What was the major finding of the study?

Most people inaccurately underestimate heights greater than 15 feet in the absence of reference points.

How does this improve population health?

Fall reported from a height of >15 feet without a reference point such as a storied building may be at risk for more significant injuries.

A notable limitation of many studies involving high-level falls is the actual measurement of the heights involved.^1^ Previous studies that focused on high-level falls used various methods of obtaining the height from which the patient fell. Some of these methods include speaking with first responders, medical chart review, or self-report by the patient or bystanders, with and without reliable height reference points, such as storied buildings.^7^,^19^

## METHODS

**Figure 2:**
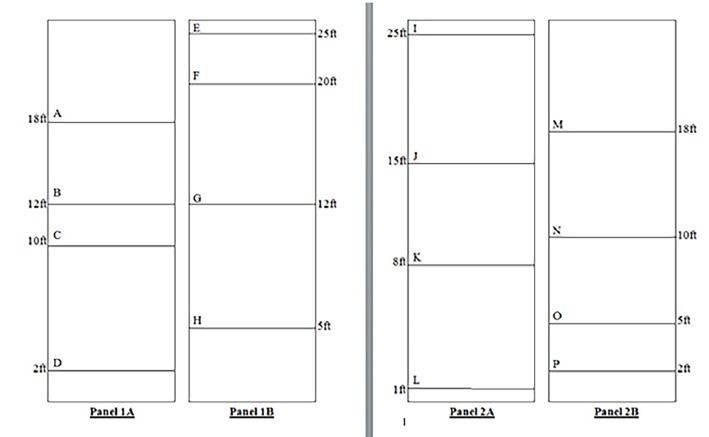
Schematic of fabric panels used by study participants to estimate height.

We recruited volunteer participants varying in age, gender, and educational background to estimate height in feet of 16 horizontal lines. Large fabric panels pre-marked with four different lines corresponding to various heights were suspended from a ceiling of an indoor site, which did not have visible stories or other reference points as confounders. The first fabric panel was labeled on one side as panel 1A, which contained four lines labeled A through D. The alternate side of this panel was labeled 1B, containing four lines labeled E through H. A second fabric panel was labeled on one side as panel 2A, containing four lines labeled I through L. The alternate site of this panel was labeled 2B, containing lines labeled M through P (Figure 2).

We created an answer key showing corresponding heights to the lines labeled A through P. This was not shared with study participants and was maintained only for data analysis. A ballot form (Appendix) was given to participants while viewing the panels. Each line, labeled A through P, had four possible answers in a multiple-choice format from which to select as an estimate.

Exclusion criteria for participants were those who could not participate for mental or physical disability, as well as those under the age of 18 years. Volunteers were given information regarding the study but not the objectives and were consented for participation. The ballot forms were sequentially numbered for purposes of tracking ballots and were otherwise anonymous. Participants were handed a ballot upon entering the room, given instructions on how to complete the survey, and the ballot was recollected upon completion.

Participants were monitored during the survey and not permitted to discuss their guesses with each other. They were positioned in the middle of the room, approximately 20 ft from the viewed panel. Participants first viewed Panel 1A and chose their answers. They then turned around to view Panel 2A and again chose their answer. They were not permitted to turn back around to look at the previous panel. While participants viewed Panel 2A, Panel 1A was pivoted to the 1B side to further prevent them from comparing heights to their previous guesses. Participants then turned around, viewed Panel 1B and chose their answers. While doing this, Panel 2A was pivoted to the 2B side. Finally, participants turned around to view Panel 2B and choose their answers. The initial goal was to obtain enrollment of 100 volunteers.

## RESULTS

**Figure 3:**
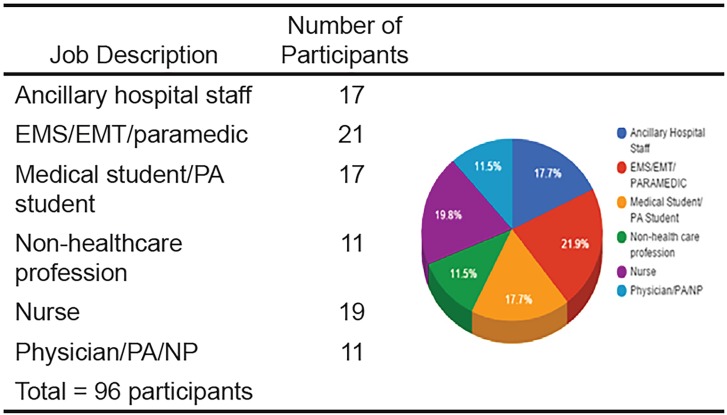
Distribution of study participants by job description. *EMS*, emergency medical service; *EMT*, emergency medical technician; *PA*, physician’s assistant; *NP*, nurse practitioner.

We enrolled a total of 96 participants. Figure 3 shows the distribution. Table 1 demonstrates the percentages of correct identifications for each line, broken down by job description (referred to as “groups”). These percentages were obtained by dividing the number of correct answers for the group by the total number of respondents in the group. Viewing these data in table form allows easy assessment for trends, showing that at lower heights, participants were more likely to correctly guess the height. For heights equal to or greater than 15 ft, less than 50% of participants in each group were able to correctly identify the height.

We further evaluated the data to reveal any trend for predisposition toward overestimating or underestimating heights when guessed incorrectly. We can determine how height (on x axis) coincides with the number of responses (on the y axis) for each subset (underestimation, overestimation, and correct). We plotted a linear function based on these data and used www.statscrunch.com to calculate the correlation coefficient (R).

We completed an a priori power analysis for the bivariate correlation using the GPower 3.0 program (Faul, Erdfelder, Lang, & Buchner, 2007). Two-tailed p values were employed. Power was set to 0.80, meaning there would be an 80% probability of reaching statistical significance if the obtained sample differences were truly present in the population. The sample size for the current study was n=96. Results from the power analysis revealed that a sample size of 29 would be sensitive to differences in ranks associated with large effect sizes (i.e., Cohen’s [1988] f = 0.50, minimal n required by the power analysis = 29). Therefore, given an obtained sample size of 96, the study is sensitive to a large effect size.

**Figure 4:**
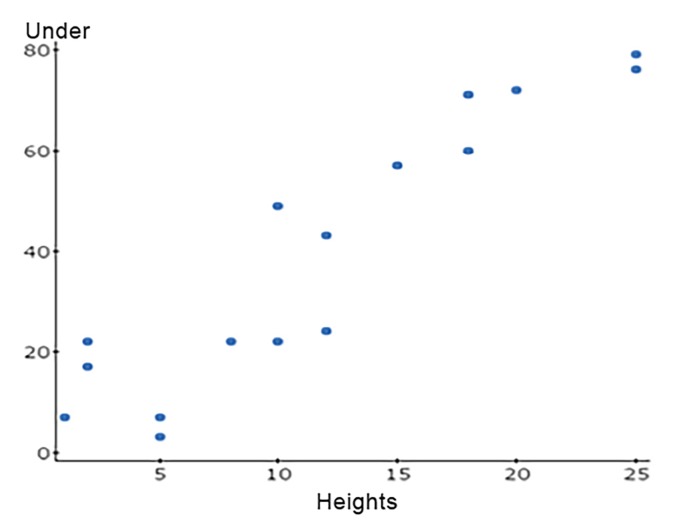
Scatter plot of heights vs. number of underestimations. For underestimation, R=+0.926, showing a strong positive correlation between the heights and number of underestimations. As the heights increased, more people consistently underestimated the correct height. Using a simple linear regression, the slope of R has a p- value=<0.001, suggesting that this trend is statistically significant. *R,* correlation coefficient.

**Figure 5:**
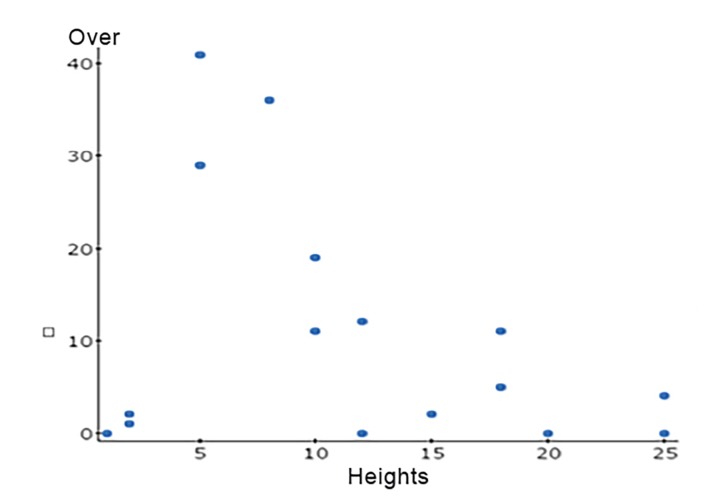
Scatter plot of heights vs. number of overestimations. For overestimation, R=−0.0331, showing a very weak negative correlation between the heights and number of overestimations. This data does not significatnly suggest that there was a prominent trend for respondents to overestimate with increasing height length,. Using a simple linear regression, the slope of R has a p-value=0.2111, suggestion that this relationship is not statistically significant. *R,* correlation coefficient.

**Figure 6:**
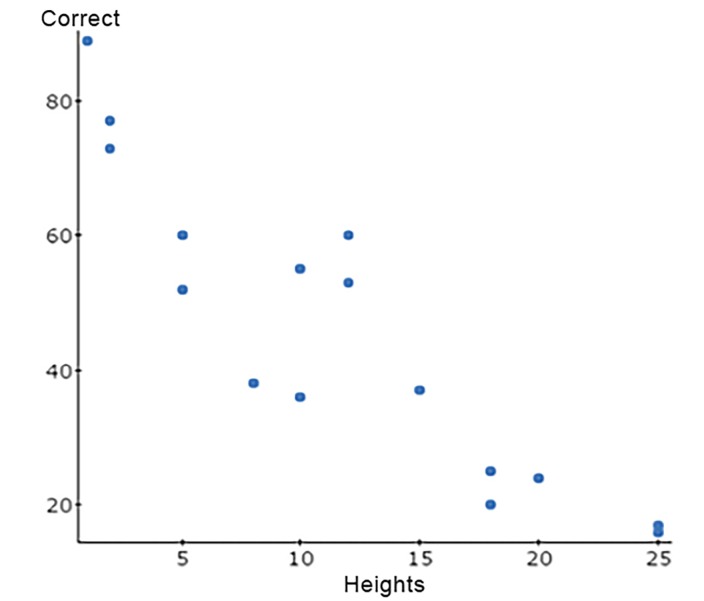
Scatter plot of heights vs number of overestimations. For correct estimation, R=−0.904 showing a strong negative correlation between the heights and number of correct responses. As the heights increased, the number of correct guesses from respondents reliably decreased. Using a linear regression, the slope of R has a p-value=<0.001, suggesting that this trend is statistically significant. *R,* correlation coefficient.

In our panel design, there were repeated heights on different panels. The goal of this design was to evaluate if participants were able to correctly identify the same height line, but on different panels with varying surrounding lines as reference points (Figures 4–6). Table 2 demonstrates the heights that were duplicated and the lines corresponding to that height. The two lines for each height are referred to as a “pair.” Each line of the pair was positioned on a panel to have either a “near” reference point or a “far” reference point. The distances between the “near” reference points and the height to be estimated ranged from 2–6 ft. The distances between the “far” reference points and the height to be estimated ranged from 5–10 ft.

We completed an a priori power analysis for the dependent samples t-test using the GPower 3.0 program (Faul, Erdfelder, Lang, & Buchner, 2007). Two-tailed *p* values were employed. Power was set to 0.80, meaning there would be an 80% probability of reaching statistical significance if the obtained sample differences were truly present in the population. The sample size for the current study was n=96. Results from the power analysis revealed that a sample size of 34 would be sensitive to differences in ranks associated with large effect sizes (i.e., Cohen’s [1988] *f* = 0.40, minimal *n* required by the power analysis = 34). Therefore, given an obtained sample size of 96, the study is sensitive to a large effect size.

We calculated the average relative error for each panel in the pair sets. A paired t-test was used to calculate if the average relative error for the “near group,” 11.5%, was a statistically significant difference from the average relative error for the “far group,” 16.4%. With a p= 0.08, this is not a statistically significant difference, meaning that the proximity of possible reference points does not consistently influence the bystander’s estimation of height.

## DISCUSSION

Acute vertical deceleration is a major cause of significant morbidity and mortality in the urban trauma setting. Velmahos et al. performed a prospective study that evaluated 187 patients who presented after a fall from a height between 5–70 ft. This study found that fractures were the most common form of injuries. Spinal cord and intra-abdominal organ injuries were also very common with falls from any height. Injuries sustained after a higher than 60 ft free-fall are usually lethal. This study concluded that the height of the fall is a good predictor of injury severity and outcome prognosis.^5^ Parreira et al. performed a retrospective study comparing the injuries sustained in falls vs. those in other mechanisms of blunt trauma, and found that those involved in falls had significantly higher rates of skeletal injuries.^20^

Multiple studies have postulated a correlation between height of fall and severity of injury.^5^–^8^ The American College of Surgeons recommends that patients injured in falls from heights greater than 20 ft (1 meter = 3.2 feet) need to be taken to a trauma center.^3^,^4^ The use of the height of a fall as a predictor of severe injury has been assessed as a part of several studies of trauma triage. Heights are factors that have been confounding in some studies.^1^ These data are often an estimation provided by prehospital personnel, first-responders, or bystanders. Many studies disclose how height values were obtained in high-level fall patients. In prospective studies performed, the height of the falls was obtained from police, fire-rescuers, paramedics, witnesses or patients themselves.^5^,^7^,^13^ Most retrospective studies assessing height injuries obtained height through medical records, chart reviews or national trauma registries.^1^,^3^,^6^,^14^,^16^ Other studies that looked at falls from building, balconies or high-level vertical fall, calculated their heights as an estimation based on how many stories each patient fell.^2^,^13^,^17^

Due to the high costs involved in healthcare spending, and in trauma team activation and work-ups in particular, it is of great interest to reduce unnecessary ordering of CTs.^22^, ^23^ Given the importance of fall height in clinical decision-making, the reliability of bystander-reported heights was investigated in this study, with the hypothesis that in the absence of a reference point such as a storied building, the estimated fall height may often be inaccurate. We found that at lower heights, participants were more likely to correctly estimate the height. In this study, the estimation was less reliable in heights greater than 15ft. Furthermore, it was found that for greater heights, inaccurate estimations were more likely to be underestimated than overestimated. Without a frame of reference, bystanders may be less accurate in estimating heights greater than 15 ft, especially in the absence of a reference point. It may be useful, then, to ask whether a storied building was nearby or if the information has validity in some other way (i.e., known heights of our machines/scaffolding/ladders). It is possible that underestimation may lead to missed injuries, and overestimation may lead to unnecessary work-up. Future studies with equal distribution of participants in each category would allow a proper analysis of variance that may reveal if one particular group of people is more accurate at estimating heights, or if additional historical factors can further vet these patients into a narrower pool in terms of work-up in protocolized trauma care. Overall, falls are a major cause of morbidity and mortality in the trauma patient and the heights estimated by those present at the scene may be inaccurate; nonetheless, it is still used as valid information pertaining to the mechanism.

## LIMITATIONS

Our study has several limitations that we must acknowledge. Previous trauma studies that assessed injuries due to high-level falls included evaluation of victims who fell from heights up to 70 ft.^5^ When planning on making fabric panels to this height, our greatest challenge was to find a location to accommodate such a height. The data collection period was between December and January, when having panels set up outdoors was a concern due to possibility of inclement weather. The location with the highest ceiling height at our institution was our hospital church, which allowed for setting up panels with the highest length of 25ft.

Our original goal was to recruit 100 prehospital respondents. Ideally, EMS participants were to be recruited at EMS stations/firehouses. However, because the logistics of assembling freestanding panels in these settings proved not to be feasible, we expanded our participant pool to include those listed in “Job Description” in Figure 6, as anyone could potentially estimate the height of a fall in the field. A degree of respondent bias must also be taken into account. Some variation in height estimation is inherent in the participant’s own height, which can alter perception of viewed heights.

Additionally, participants were observed looking at their fellow co-participants, using the perceived height of their co-participant to estimate the height of the line on the panel. As each group varied in participants, this may have altered some of the participants’ perceptions. Other limitations include number of respondents; if we could have had a greater power in the study, there might have been more noticeable differences in height accuracy between first responders vs. non-first responders. Also, we did not include age, which may also play a role in accuracy. Finally, estimating heights in a vacuum is not how it actually occurs in real life. Our aim was to determine accuracy in estimation in the absence of buildings; in an actual setting, this would include falls from a large piece of machinery, tree, or other structure without a clear height-reference point.

## CONCLUSION

This small study from a community hospital showed that bystanders may not estimate heights accurately in the field. The greater the reported height, the less likely it is to be accurate. Additionally, there is a higher likelihood that falls from greater than 15 ft may be underestimated. Further studies are warranted to determine additional demographic and environmental factors that may affect the accuracy of bystander reports in the mechanism of traumatic injuries. Potential bystanders are more likely to underestimate the actual height of a fall. High-level falls are linked to more life-threatening injuries; therefore, it may be prudent to assume a more severe mechanism than inferred from the height provided via bystander report.

## Supplementary Information



## Figures and Tables

**Table 1:** Percentage of correct answers per line divided by job description.

	A (18ft)	B (12ft)	C (10ft)	D (2ft)	E (25ft)	F (20ft)	G (12ft)	H (5ft)	I (25ft)	J (15 ft)	K (8ft)	L (1ft)	M (18ft)	N (10ft)	O (5ft)	P (2ft)
Ancillary hospital staff	11.8	64.7	52.9	82.4	17.6	17.6	58.8	52.9	23.5	35.3	29.4	94.1	29.4	47.1	47.1	82.4
EMS/EMT/paramedic	23.8	61.9	61.9	71.4	19.0	28.6	61.9	61.9	19.0	47.6	33.3	90.5	33.3	52.4	76.2	61.9
Medical student/PA student	23.5	82.4	58.8	82.4	35.3	35.3	52.9	58.8	17.6	35.3	52.9	94.1	17.6	23.5	82.4	82.4
Non-healthcare profession	36.4	45.5	45.5	81.8	9.1	27.3	36.4	36.4	18.2	45.5	63.6	81.8	0.0	18.2	36.4	63.6
Nurse	42.1	63.2	57.9	73.7	5.3	10.5	47.4	52.6	10.5	26.3	36.8	94.7	5.3	31.6	47.4	78.9
Physician/PA/NP	27.3	45.5	63.6	100	9.1	36.4	72.7	54.5	18.2	45.5	27.3	100	36.4	45.5	81.8	90.9

*ft*, feet; *EMS,* emergency medical service; *EMT*, emergency medical technician; *PA,* physician’s assistant; *NP,* nurse practitioner.

**Table 2:** Lines assigned to “near” or “far” groups.

Height (feet)	Near	Far
2	P	D
5	O	H
10	C	N
12	B	G
18	A	M
25	E	I
