# Supplementary figures and images for: Accuracy of Height Estimation Among Bystanders
Source: West J Emerg Med. 2018 Jul 26;19(5):813–9. doi: 10.5811/westjem.2018.5.34877 (PMC6123090; doi:10.5811/westjem.2018.5.34877)

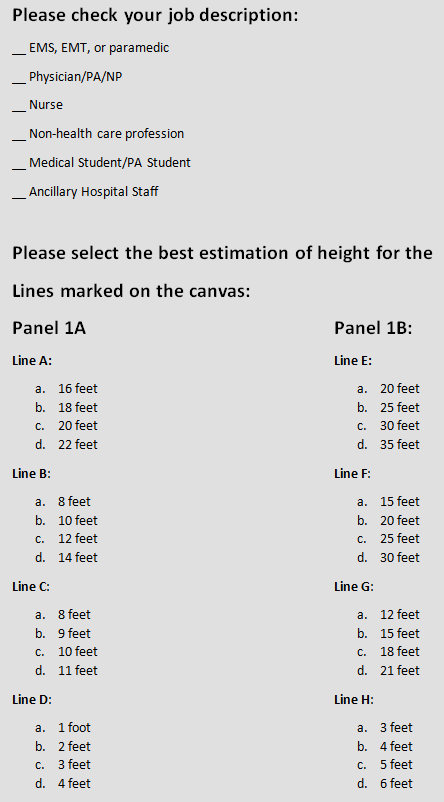
**Appendix.** Ballot and form.


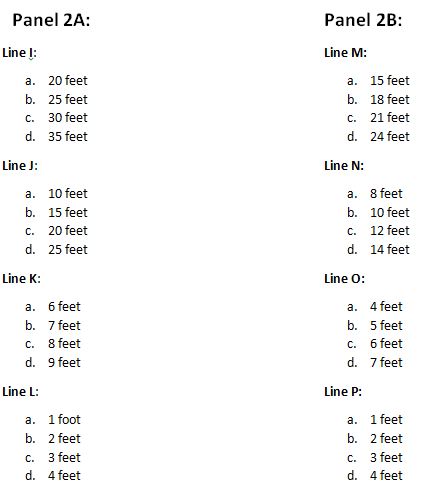

Supplement: Supplementary file 1 [file wjem-19-813-s001.docx]
